# Analysis of *RET* promoter CpG island methylation using methylation-specific PCR (MSP), pyrosequencing, and methylation-sensitive high-resolution melting (MS-HRM): impact on stage II colon cancer patient outcome

**DOI:** 10.1186/s13148-016-0211-8

**Published:** 2016-04-26

**Authors:** Muriel X. G. Draht, Kim M. Smits, Valérie Jooste, Benjamin Tournier, Martijn Vervoort, Chantal Ramaekers, Caroline Chapusot, Matty P. Weijenberg, Manon van Engeland, Veerle Melotte

**Affiliations:** Department of Pathology, GROW - School for Oncology & Developmental Biology, Maastricht University Medical Center, P.O. Box 616, 6200 MD Maastricht, The Netherlands; Department of Radiation Oncology (MAASTRO), GROW - School for Oncology and Developmental Biology, Maastricht University Medical Center, Maastricht, The Netherlands; Registre Bourguignon des cancers digestifs, INSERM U866, Universite de Bourgogne, Centre Hospitalier Universitaire de Dijon, Dijon, France; Service de Pathologie, Centre Hospitalier Universitaire de Dijon, Dijon, France; Zuyd University of Applied Sciences, Heerlen, The Netherlands; Chemelot Innovation and Learning Labs, Geleen, The Netherlands; Department of Epidemiology, GROW - School for Oncology and Developmental Biology, Maastricht University Medical Center, Maastricht, The Netherlands

**Keywords:** DNA methylation, RET, Clinical sensitivity, Analytic sensitivity, MSP, pyrosequencing, MS-HRM

## Abstract

**Background:**

Already since the 1990s, promoter CpG island methylation markers have been considered promising diagnostic, prognostic, and predictive cancer biomarkers. However, so far, only a limited number of DNA methylation markers have been introduced into clinical practice. One reason why the vast majority of methylation markers do not translate into clinical applications is lack of independent validation of methylation markers, often caused by differences in methylation analysis techniques. We recently described *RET* promoter CpG island methylation as a potential prognostic marker in stage II colorectal cancer (CRC) patients of two independent series.

**Methods:**

In the current study, we analyzed the *RET* promoter CpG island methylation of 241 stage II colon cancer patients by direct methylation-specific PCR (MSP), nested-MSP, pyrosequencing, and methylation-sensitive high-resolution melting (MS-HRM). All primers were designed as close as possible to the same genomic region. In order to investigate the effect of different DNA methylation assays on patient outcome, we assessed the clinical sensitivity and specificity as well as the association of *RET* methylation with overall survival for three and five years of follow-up.

**Results:**

Using direct-MSP and nested-MSP, 12.0 % (25/209) and 29.6 % (71/240) of the patients showed *RET* promoter CpG island methylation. Methylation frequencies detected by pyrosequencing were related to the threshold for positivity that defined *RET* methylation. Methylation frequencies obtained by pyrosequencing (threshold for positivity at 20 %) and MS-HRM were 13.3 % (32/240) and 13.8 % (33/239), respectively. The pyrosequencing threshold for positivity of 20 % showed the best correlation with MS-HRM and direct-MSP results. Nested-MSP detected *RET* promoter CpG island methylation in deceased patients with a higher sensitivity (33.1 %) compared to direct-MSP (10.7 %), pyrosequencing (14.4 %), and MS-HRM (15.4 %). While *RET* methylation frequencies detected by nested-MSP, pyrosequencing, and MS-HRM varied, the prognostic effect seemed similar (HR 1.74, 95 % CI 0.97–3.15; HR 1.85, 95 % CI 0.93–3.86; HR 1.83, 95 % CI 0.92–3.65, respectively).

**Conclusions:**

Our results show that upon optimizing and aligning four *RET* methylation assays with regard to primer location and sensitivity, differences in methylation frequencies and clinical sensitivities are observed; however, the effect on the marker’s prognostic outcome is minimal.

**Electronic supplementary material:**

The online version of this article (doi:10.1186/s13148-016-0211-8) contains supplementary material, which is available to authorized users.

## Background

DNA methylation has become an attractive target for biomarker research. It can be used as a marker for early detection of a disease or to predict prognosis or response to therapy. Although many researchers focus on the identification of novel methylation DNA markers and improvement of sensitivity and specificity of markers, currently, the applicability of methylation markers in the clinic is very limited. Of the hundreds of putative methylation markers reported in literature, only few are currently used in clinical practice. *NDRG4* and *BMP3* promoter CpG island methylation analysis is part of the FDA-approved molecular marker assay named Cologuard for early detection of colorectal cancer (CRC) [1, 2]. *SEPT9* is a blood-based alternative for colorectal cancer diagnosis [3]. *GSTP1*, *RASSF1*, and *APC* are part of a biopsy tissue-based, multiplex methylation-specific PCR (MSP) assay for prostate cancer detection [4, 5]. A urine-based DNA-methylation assay profiling *TWIST1* and *NID2*, in combination with non-epigenetic markers, has shown to be useful for bladder cancer diagnosis in hematuria patients [6].

The absence of DNA methylation markers in clinical settings is mainly due to an ill-powered marker identification strategy in small selected series resulting in chance findings and false-positive identification of biomarkers. In addition, a lack of adequate validation of markers in independent patient series generating a good level of evidence [7–9], and the use of technologies with varying sensitivity and specificity to analyze these markers, leads to discrepant results [10, 11]. With the development of diverse DNA methylation analysis techniques, comparison of methylation data has become more complex. Substantial variation in frequency and clinical value of specific methylation markers has been reported [12–14]. The same holds true for the frequency of the CpG island methylator phenotype (CIMP). The absence of a universal definition to define CIMP has resulted in challenging interpretation of the data, as well as studies showing a lack of validation [15].

In order to allow a better comparison of methylation data, interpretation of the clinical relevance, and, ultimately, implementation of methylation markers into clinical practice, more effort is required to better take into account the analytical and clinical sensitivity and specificity of a specific assay. A large variety in the quantification of methylation signals exists in published DNA methylation marker studies. For example, variation in primer location and design and threshold values for determining test positivity affect sensitivity, specificity, and ultimately the clinical relevance of a biomarker [16, 17].

MSP, pyrosequencing, and methylation-sensitive high-resolution melting (MS-HRM) are widely used techniques to assess DNA methylation (Table 1). MSP, developed in 1996 [18], is a sensitive, relatively simple, and inexpensive technique, which made it an attractive tool to study DNA methylation in the past decades. MSP is based on PCR primers, which can distinguish unmethylated and methylated DNA. The unmethylated primers will only amplify sodium bisulfite-converted DNA in the unmethylated condition, while the methylated primers are specific for sodium bisulfite-converted methylated DNA. This technology provides information regarding the methylation status of the genomic region covered by the MSP primers. However, suboptimal primer design and optimization of the assay can lead to non-reproducible results [8, 19, 20], and the high sensitivity of the technique has led to so-called false-positive interpretation of results [21]. Uhlmann et al. first described detection of DNA methylation by pyrosequencing in 2002 [22]. This technique is based on the detection of emitted light, which is generated when a nucleotide gets incorporated in the PCR template by DNA polymerase, activating an enzymatic cascade. In the presence of adenosine triphosphate (ATP) sulfurylase, released pyrophosphate will be converted to ATP, which in turn is an energy source for the oxidization of luciferin. This reaction generates a light signal. The amount of incorporated nucleotides is proportional to the amount of emitted light [23, 24]. Due to the difference in DNA sequence of bisulfite-converted DNA, pyrosequencing enables quantification of DNA methylation. Wojdacz and Dobrovic were the first to describe the principle of MS-HRM in 2007 [25]. This technique is based on the detection of a fluorescent signal, which is generated by a dye that incorporates in double-stranded DNA which is released upon melting, and which can detect single nucleotide differences based on small deviations in melting temperatures [25]. In order to study the methylation of a DNA sample, unmethylated and methylated references have to be included. A difference in the hydrogen bonds of methylated CpG dinucleotides and unmethylated CpG dinucleotides results in a different melting profile with methylated DNA melting at a higher temperature compared to unmethylated DNA [26].

**Table 1:** Characteristics of different methylation analysis techniques and reported recommendations for assay design

	Pyrosequencing	MSP	MS-HRM
Material	FF preferred	FFPE/FF	FF preferred
Closed-tube			√
€/sample	4.76	1.21–1.64^b^	1.76
Amplicon length (bp)	<400^a^/<200 bp	<160	<200
Annealing temp. (°C)	50–68	56^a^/60–66	58–66
Number of CpG dinucleotides included in primer	1 or less/none	1 or less^a^/3 or more	2 or less
Analysis of CpG dinucleotides	CpG dinucleotides in between primers	CpG dinucleotides covered by primers	CpG dinucleotides covered and in between primers

*MSP* methylation-specific PCR, *MS-HRM* methylation-sensitive high-resolution melting, *FFPE* formalin-fixed paraffin-embedded, *FF* fresh frozen

^a^Conditions of preamplification PCR (in case of nested-MSP: flank primers)

^b^Direct-MSP and nested-MSP

Using MSP and pyrosequencing, we recently identified *RET* promoter methylation as a possible prognostic marker for tumor-node-metastasis (TNM classification) stage II CRC patients. Promoter methylation of *RET* was associated with a poorer prognosis in two of three independent tissue sample series using both techniques [27].

**Fig. 1:**
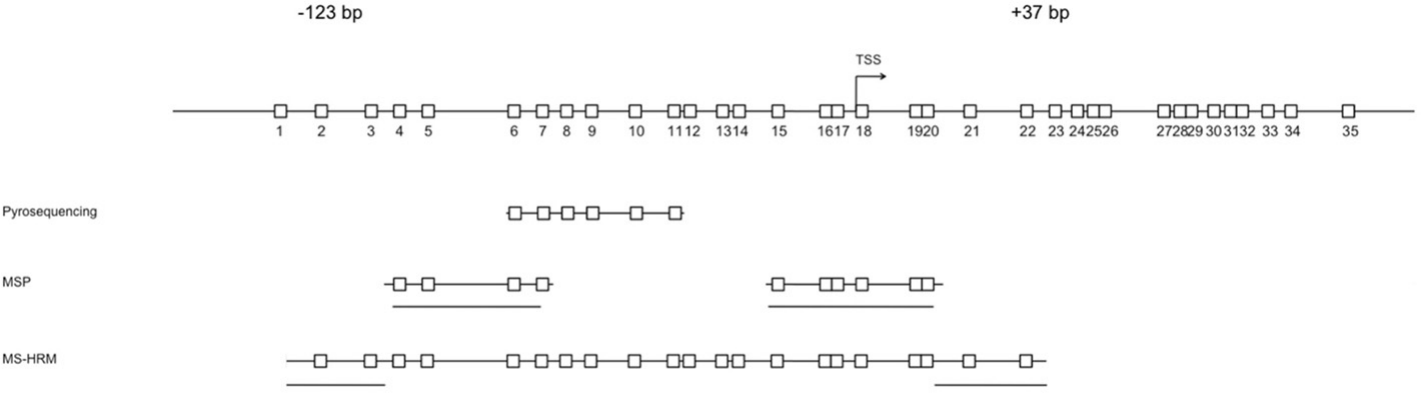
Schematic representation of the *RET* promoter CpG island (according to the EMBOSS database; located on chromosome 10q11.2) and the genomic region of primer location. Each *rectangle* represents a CpG dinucleotide. CpG dinucleotides, which are *underlined*, point to the primer locations for all four methylation analysis techniques. Direct-MSP and nested-MSP (here: MSP) share the same primers specific for methylated, as well as not methylated, DNA. Flanking primers used for nested-MSP amplify bisulfite-treated DNA, without discriminating between methylated and not methylated DNA. Therefore, these primers are not included in this figure

In this study, we analyze *RET* promoter CpG island methylation using four DNA methylation detection techniques (direct-MSP, nested-MSP, pyrosequencing, and MS-HRM) and primers aligned as much as possible regarding the genomic region (Fig. 1), to determine the influence of the test on the outcome of stage II colon cancer patients.

## Results

### Frequency and overlap of promoter CpG island methylation measured by direct-MSP, nested-MSP, pyrosequencing, and MS-HRM

Of 209 samples successfully amplified with direct-MSP primers, 25 samples (12 %) showed *RET* promoter CpG island methylation. Using nested-MSP, *RET* promoter CpG island was methylated in 71 of 240 (29.6 %) samples, confirming that nested-MSP is more sensitive than direct-MSP (Table 2). By pyrosequencing, the frequency of *RET* promoter CpG island methylation was dependent on the threshold for positivity that was used. Using a threshold for positivity of 20 % (previously defined as the most optimal threshold for positivity to predict prognosis in stage II CRC patients using *RET* methylation [27]), we detected *RET* promoter CpG island methylation in 32 out of 240 (13.3 %) patient samples. In addition, methylation frequencies were calculated for thresholds for positivity of 5, 10, 15, and 25 %. As expected, the higher the methylation threshold for positivity, the lower was the methylation frequency (Table 2). *RET* promoter CpG island methylation detected by pyrosequencing with a threshold for positivity of 5 % or higher was observed in 62 out of 240 samples (25.8 %), whereas a threshold for positivity value of 25 % decreased the methylation frequency to 10.4 % (25/240). Using MS-HRM, *RET* promoter CpG island methylation was observed in 13.8 % of 33 out of 239 samples (Table 2).

**Table 2:** Methylation frequencies of *RET* promoter CpG island methylation

	Direct-MSP	Nested-MSP	Pyroseq. 5 %	Pyroseq. 10 %	Pyroseq. 15 %	Pyroseq. 20 %	Pyroseq. 25 %	MS-HRM
Frequency of methylation in % (*n*)	12.0 (25/209)	29.6 (71/240)	25.8 (62/240)	17.1 (41/240)	15.4 (37/240)	13.3 (32/240)	10.4 (25/240)	13.8 (33/239)
HR for 3-year follow-up (95 % CI)	1.52 (0.66–3.52)	1.74 (0.97–3.15)	1.13 (0.60–2.11)	1.53 (0.78–2.97)	1.59 (0.80–3.16)	1.85 (0.93–3.86)	1.57 (0.97–3.44)	1.83 (0.92–3.65)
HR for 5-year follow-up (95 % CI)	1.13 (0.51–2.53)	1.21 (0.72–2.04)	0.89 (0.51–2.57)	1.14 (0.62–2.09)	1.20 (0.63–2.26)	1.46 (0.77–2.76)	1.34 (0.65–2.76)	1.44 (0.76–2.72)

*MSP* methylation-specific PCR, *Pyroseq.* pyrosequencing, *MS-HRM* methylation-sensitive high-resolution melting, *N* number of cases, *HR* hazard ratio, *CI* confidence interval

**Table 3:** Clinical sensitivity and specificity for methylation detection using overall survival as end point

	Sensitivity (%)	Specificity (%)
Direct-MSP	10.7	86.5
Nested-MSP	33.1	73.8
Pyrosequencing 5 % threshold	22.8	73.1
Pyrosequencing 10 % threshold	17.8	83.6
Pyrosequencing 15 % threshold	16.1	85.3
Pyrosequencing 20 % threshold	14.4	87.7
Pyrosequencing 25 % threshold	11.0	90.2
MS-HRM	15.4	87.7

Sensitivity was calculated as a/(a + b). Specificity was calculated as d/(c + d)

*MSP* methylation-specific PCR, *MS-HRM* methylation-sensitive high-resolution melting

**Fig. 2:**
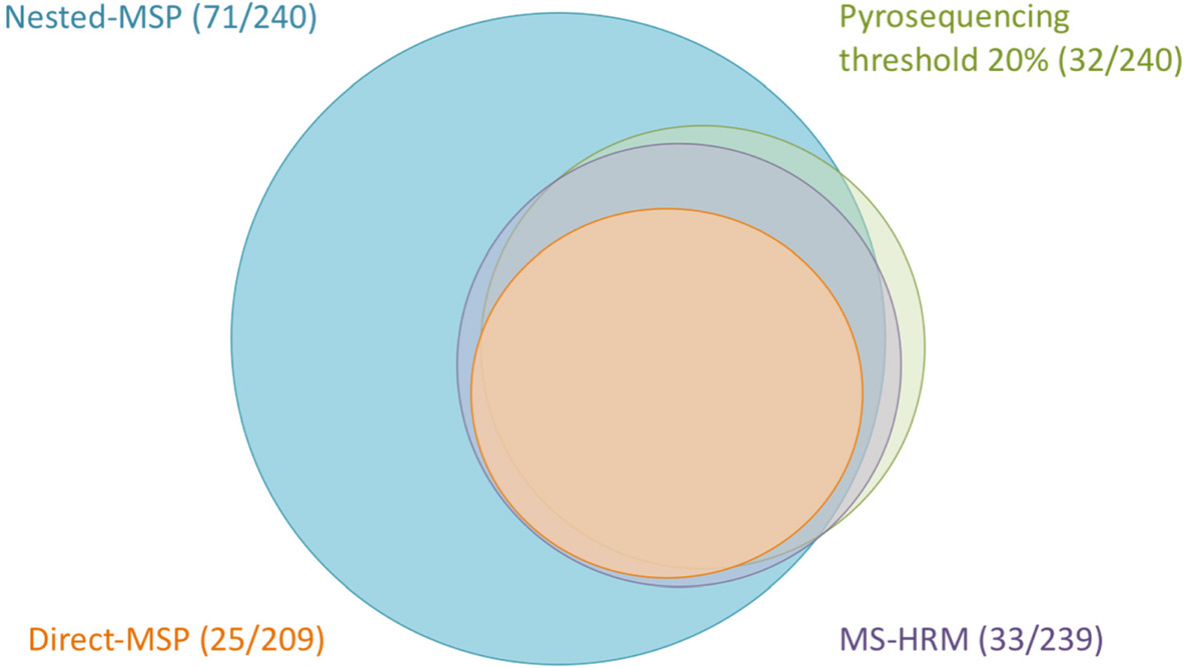
Venn diagram showing the overlap between *RET* methylation detected by direct-MSP (*orange*), nested-MSP (*blue*), pyrosequencing (*green*), and MS-HRM (*purple*)

Next, we compared the overlap between methylation analysis techniques. Direct-MSP showed a 100 % concordance with nested-MSP and MS-HRM in that all 25 methylated samples as determined by direct-MSP were also methylated using nested-MSP and MS-HRM (Fig. 2). Of these 25 samples, 92 % (*n* = 23) were also methylated using pyrosequencing (positivity threshold 20 %). None of the 209 samples was exclusively detected as methylated by using direct-MSP. For nested-MSP, the overlap with direct-MSP (100 % overlap), pyrosequencing (93.8 % overlap), and MS-HRM (97 % overlap) was high, although nested-MSP also detected *RET* methylation, where direct-MSP, pyrosequencing, and MS-HRM did not. This was the case for 63.6 % (42/66), 57.7 % (41/71), and 54.3 % (38/70) of samples. Out of 71 samples methylated by nested-MSP, 23 samples (32.4 %) were methylated by all four methylation analyses techniques (Fig. 2). We observed that pyrosequencing with a threshold for positivity of 20 % and MS-HRM showed the best overlap. Of the 32 samples methylated by pyrosequencing and 33 by MS-HRM, 30 samples were methylated by both techniques. Although nested-MSP and pyrosequencing with a threshold for positivity of 5 % showed comparable methylation frequencies (29.6 and 25.8 %, respectively), methylation was not always present in the same samples. Thirty-nine (54.9 %) out of 71 methylated samples by nested-MSP and 39 (62.9 %) out of 62 methylated samples by pyrosequencing with a threshold for positivity of 5 % (62.9 %) showed concordance in *RET* methylation using these two assays.

### Predictive value of *RET* promoter CpG island methylation measured by direct-MSP, nested-MSP, pyrosequencing, and MS-HRM

The high methylation frequency observed by nested-MSP and pyrosequencing using a low threshold for positivity is in literature often referred to as a false-positive detection of methylation. The question remains whether these samples are true false-positive results or whether these sensitive techniques detected a small amount of *RET* promoter CpG island methylation that is biologically and clinically relevant. Therefore, we calculated the clinical sensitivities and clinical specificities of each methylation analysis assay. Moreover, we examined whether different methylation assays influence the predictive value of *RET* promoter CpG island methylation.

For direct-MSP, the clinical sensitivity was 10.7 %, whereas the clinical specificity was 86.5 % (Table 3). The highest clinical sensitivity with 33.1 % was observed for nested-MSP. This was at the cost of a lower clinical specificity, as compared to other assays, which was 73.8 %. As for pyrosequencing, the clinical sensitivity decreased with a higher threshold for positivity. For methylation detected using a threshold for positivity at 20 %, the clinical sensitivity was 14.4 % and clinical specificity was 87.7 %. Notably, the pyrosequencing assay using a 25 % threshold for positivity showed the highest clinical specificity (90.2 %), as compared to other assays. The MS-HRM assay detected *RET* promoter CpG island methylation in deceased patients with a clinical sensitivity of 15.4 %, whereas the clinical specificity was 87.7 %. For pyrosequencing (threshold for positivity at 5 %) on the other hand, clinical specificity (73.1 %) was similar to that of nested-MSP (73.8 %); however, the clinical sensitivity was almost 10 % less than nested-MSP (22.8 and 33.1 %, respectively). Thus, although both assays detect high frequencies of *RET* methylation, the clinical sensitivity, and thus the ability of detecting patients at higher risk of dying, is markedly different, however, still relatively low for both techniques. Subgroup analysis for gender and sub-location of the tumor does not alter the conclusions (data not shown).

**Fig. 3:**
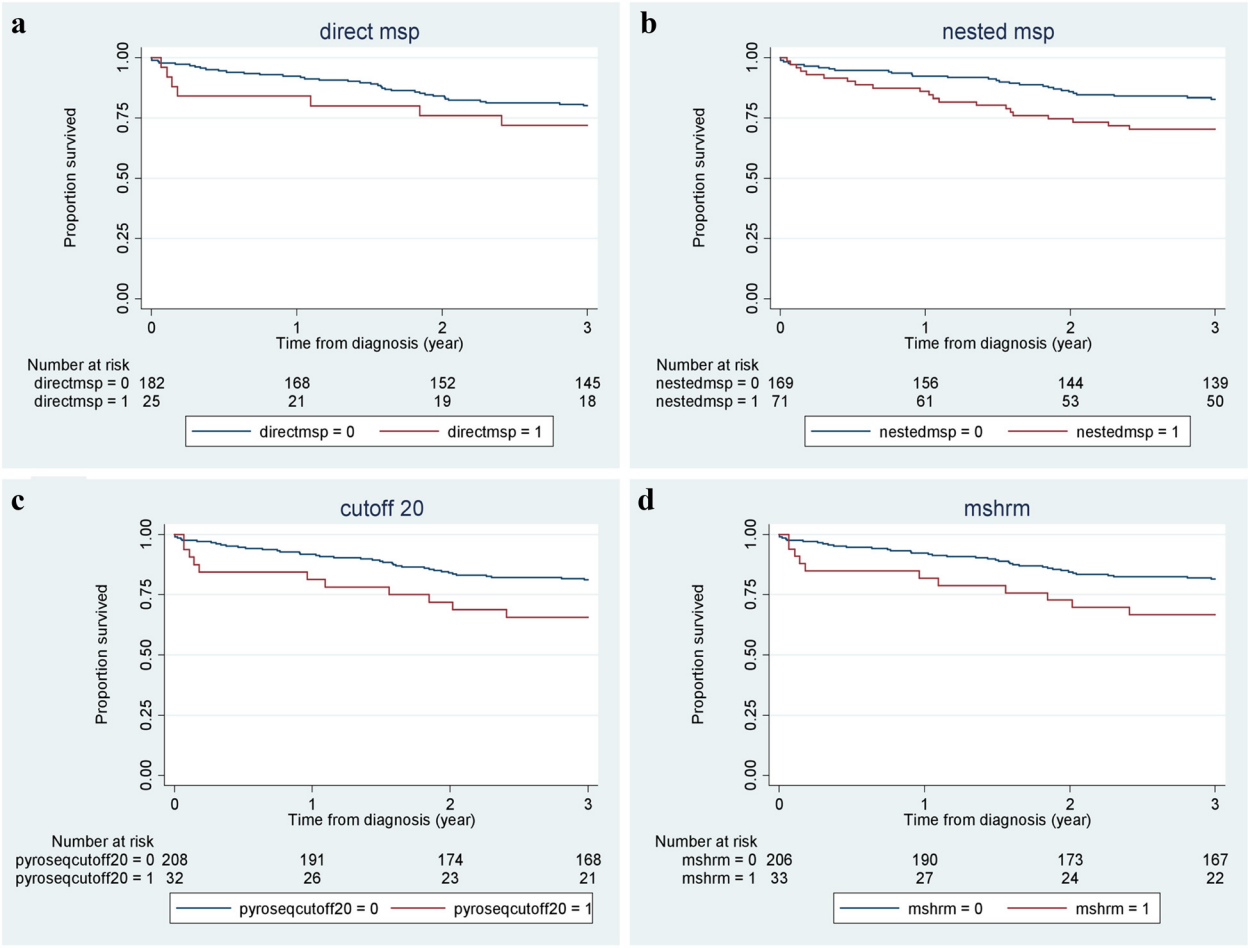
Kaplan-Meier curves of overall survival for three years of follow-up. Patients with *RET* methylation (*red line*) measured by direct-MSP (**a**), nested-MSP (**b**), pyrosequencing with a threshold for positivity at 20 % (**c**), and MS-HRM (**d**) showed a trend towards a poorer prognosis; however, this was not statistically significant (*p* > 0.05)

In order to determine whether the prognostic value of *RET* promoter CpG island methylation is affected by the method of analysis, we performed survival analyses for each assay. The results of Cox proportional hazard analyses are summarized in Table 4. While none of the hazard ratios (HRs) are statistically significant in this series of patients, a trend towards a poorer prognosis with *RET* methylation can be seen from the Kaplan-Meier curves (Fig. 3) for nested-MSP and pyrosequencing (threshold for positivity at 20 %) as well as MS-HRM. Also, corresponding HRs were in similar range (HR 1.74, 95 % confidence interval (CI) 0.97–3.15; HR 1.85, 95 % CI 0.93–3.86; HR 1.83, 95 % CI 0.92–3.65, respectively; Table 4).

**Table 4:** Baseline characteristics of the study population

Total *N* (%)	240 (100)
Age at diagnosis	71.6
Gender	
Female	101 (42.8)
Male	139 (57.9)
Sub-location tumor	
Proximal	109 (45.4)
Distal	87 (36.6)
Rectosigmoid	44 (18.3)

## Discussion

DNA methylation markers have been recognized as promising diagnostic, prognostic, and predictive tools for decades. However, lack of sufficient validation and evaluation of markers and lack of consensus regarding the best technique to detect methylation markers have prevented the introduction of the vast majority of methylation markers into daily clinical practice. A variety of DNA methylation analysis techniques have been developed to analyze genome-wide methylation and methylation of candidate genes/DNA regions. Each technology has its own test characteristics, based on primer and probe design, amplicon size, PCR conditions (for example, number of amplification cycles), analytical sensitivity and specificity, and interpretation of results. These differences in test characteristics make it hard to objectively compare the different methods, which is especially true for techniques that analyze candidate genes/DNA regions.

Recently, *RET* has been described as a candidate tumor suppressor gene in CRC, and we have shown that promoter CpG island methylation of *RET* is associated with tumorigenesis and a poorer prognosis of CRC patients in several independent patient populations [27, 28].

Despite the above mentioned challenges in comparing DNA methylation assays, we set out to compare direct-MSP, nested-MSP, pyrosequencing, and MS-HRM to qualitatively and quantitatively analyze *RET* promoter CpG island methylation and its predictive value in stage II colon cancer patients. In order to do this, we aligned the assays as much as possible regarding the genomic region, by designing primers for each *RET* promoter CpG island methylation assay as close as possible to the genomic region of the primer sets that have been associated with the outcome in our previous study [27].

This comparison showed that, as expected, direct-MSP is less sensitive than nested-MSP and that methylation frequencies detected by pyrosequencing were related to the threshold for positivity that defined *RET* methylation. We also showed that the concordance between the methylation detection varies between the different techniques (using the primers as depicted in Fig. 1) and is as low as 54.9 % for nested-MSP versus pyrosequencing (threshold for positivity at 5 %) and as high as 100 % for direct-MSP versus nested-MSP. Although methylation detected by MSP has often been described as a false-positive methylation, nested-MSP detected *RET* promoter CpG island methylation in deceased patients with a higher clinical sensitivity (33.1 %) when compared to direct-MSP (10.7 %), pyrosequencing with a threshold of positivity of 20 % (14.4 %), and MS-HRM (15.4 %). Higher thresholds for positivity to determine methylation for pyrosequencing showed lower clinical sensitivities, whereas the clinical specificity was better for quantitative techniques as compared to nested-MSP. We can argue that the clinical specificity of nested-MSP might indicate an overestimation of methylation in patients with a good prognosis (*RET* methylation 23 %). However, as for pyrosequencing (threshold for positivity at 20 and 25 %) and MS-HRM, which showed good clinical specificities and good methylation overlap, the clinical sensitivities also indicate that those assays still miss a lot of deceased patients. These data indicate that samples, which were exclusively methylated by nested-MSP and not by other techniques, should not solely be considered as false-positive test results. Nonetheless, it should be kept in mind that nested-MSP was designed for detection of low levels of DNA methylation, as, for example, seen in subclones of cancer cells or tissue with a low amount of tumor cells. The relevance of small amounts of DNA methylation should be carefully interpreted and can be different between sample sources, for example, in blood and stool samples versus resection material. Our data also show that qualitative techniques such as direct-MSP and nested-MSP (although showing different methylation frequencies) can, when carefully developed, optimized, and interpreted, yield comparable clinical results as pyrosequencing and MS-HRM and could therefore be used for biomarker detection/validation. As gel-based assays are not the method of choice for clinical tests, quantitative techniques, such as closed-tube techniques, are preferred in clinical settings, which require the objective establishment of a threshold for positivity. Unfortunately, such an objective assessment and clear description of the threshold for positivity is often lacking in DNA methylation marker studies. Moreover, quantification of methylation signals is highly dependent on the percentage of tumor cells in a given specimen. However, reliable assessment of the tumor cell count is currently not a standard procedure in clinical settings, and in addition, the percentage of tumor cells estimated on hematoxylin and eosin stained slides is often not accurate [29].

It highly depends on the objective of the study, whether a high clinical sensitivity or a high clinical specificity is desired. Using different thresholds for positivity for pyrosequencing can enable the variation in assay performance by means of clinical sensitivities and specificities. This is not possible for nested-MSP that only allows a yes/no assessment of DNA methylation, which should be kept in mind preceding formulating objectives of a biomarker study. Moreover, the clinical value of a methylation marker not only depends on the test characteristics but also on clinical end points. In the current study, we only had data available on patient survival. It would be interesting to correlate the outcome of the different techniques with the recurrence of disease as end point and to assess comorbidities of these patients, as more than 50 % of CRC patients suffer from comorbidities [29].

*RET* methylation has previously been described as a possible prognostic marker for stage II CRC [27]. In the current study, the prognostic effect of methylation measured by all assessed techniques was not as evident as in the previously reported study; however, a trend towards a poorer prognosis was observed. This was most likely due to a smaller sample size as compared to the previous study. We realize that additional molecular or histology markers should be considered in combination to *RET* methylation in order to enhance the predictive value for accurate and robust identification of high-risk stage II colon cancer patients or that other markers might better predict prognosis. Nonetheless, the goal of this study was not to validate the biomarker potential of *RET* methylation but to compare the prognostic value of *RET* methylation by comparing four different techniques to assess methylation. Our results show, for the first time, that effects on overall survival did not differ despite differences in assays to measure DNA methylation and differences in clinical sensitivities and specificities.

## Conclusions

Due to the high analytic sensitivity of nested-MSP, we were able to detect more deceased patients (higher clinical sensitivity) than using direct-MSP, pyrosequencing, and MS-HRM. When selecting methylation assays for biomarker research, the choice of the methylation detection method highly depends on the objective of the study and if a high clinical sensitivity or a high clinical specificity is desired.

## Methods

### Patient samples

We used fresh frozen TNM stage II colon adenocarcinoma tissue, which was provided by the Pathology Department of the academic hospital in Dijon and which was collected from three pathology laboratories covering the area: Dijon University Hospital, Comprehensive Cancer Centre, and a private center as described previously [27, 30]. Baseline characteristics of this study population are provided in Table 4.

### DNA isolation and sodium bisulfite conversion

DNA was isolated using the Nucleospin®96 tissue kit (Macherey-Nagel, Düren, Germany) as previously described [31]. DNA was available for 241 stage II CRC patients to perform direct-MSP, nested-MSP, pyrosequencing, and MS-HRM.

Sodium bisulfite modification of genomic DNA was performed using the EZ DNA methylation kit (ZYMO Research Corporation, Orange, CA, USA) according to the manufacturer’s instructions.

### Detection of DNA methylation

In order to compare the test performance of four different methylation detection techniques (direct-MSP, nested-MSP, pyrosequencing, and MS-HRM) for *RET* CpG island methylation, we aimed to design the assays within the same genomic region as close as possible to each other (Fig. 1). Nevertheless, due to the CpG dinucleotide-rich region and the technical differences between the assays, analysis of identical CpG dinucleotides was not always possible. As shown in Fig. 1, direct-MSP and nested-MSP measure methylation of the CpG dinucleotides covered by the primers, whereas MS-HRM detects methylation of CpG dinucleotides in between the forward and reverse primer as well. As for pyrosequencing, sequencing primer design is desired without covering CpG dinucleotides, while the CpG dinucleotides in between the primers are quantified for methylation.

#### Direct-MSP and nested-MSP

In this study, we performed direct-MSP, and the more sensitive variant, nested-MSP, which has been developed specifically to amplify DNA obtained from formalin-fixed, paraffin-embedded tissue [18, 32]. Primer pairs were designed near the putative transcriptional start site (TSS; Fig. 1; Additional file 1: Table S1). For nested MSP, the genomic region of interest is amplified with flanking primers prior to discriminating with primers specific for methylated and unmethylated DNA. PCR amplification data were analyzed by using gel electrophoresis. Of the 241 analyzed samples, direct-MSP did not show a result for 32/241 (13.3 %) of the samples, whereas for nested-MSP, one sample (0.4 %) did not show a result.

#### Pyrosequencing

For the current study, pyrosequencing was performed as described previously using the PyroMark Q24 kit (Qiagen, Hilden, Germany) [27]. Briefly, PCR reactions were carried out in a 25 μL final volume comprising 12.5 μL of PyroMark Master Mix, 2.5 μL of CoralLoad buffer, 0.5 μL of forward and biotinylated reverse primers (0.2 μM final concentration), 8 μL of RNase-free water, and 1 μL of bisulfite-treated DNA (20 ng). The biotinylated PCR products were processed as described elsewhere [31]. Results were analyzed using PyroMark Q24 2.0.6 software. To ensure successful bisulfite conversion of unmethylated cytosines, an internal conversion control that corresponded to the position of a non-CG cytosine (not subject to methylation) was present in the dispensation sequence. Of the 241 samples analyzed, one sample (0.4 %) did not show a result. The sequences for the genomic region of pyrosequencing primers are provided in Additional file 1: Table S1.

#### MS-HRM

The MS-HRM assay was designed as close as technically possible to the genomic region being analyzed by direct-MSP, nested-MSP, and pyrosequencing assays (Fig. 1). The sequences for the genomic region of MS-HRM primers are provided in Additional file 1: Table S1. By including a CpG dinucleotide in each primer, we aimed to reduce the possibility of PCR bias [25]. PCR amplification was carried out in a total volume of 20 μL of Precision Melt Supermix (Biorad), 250 nM of forward and reverse primer, and 25 ng of bisulfite-treated DNA template. Amplification was performed on a LightCycler 96 (Roche) and consisted of 2 min at 95 °C, followed by 70 cycles of 10 s at 95 °C, 30 s at 56 °C, and 30 s at 72 °C. According to the manufacturer’s recommendations, HRM analysis spanned a temperature range from 65–95 °C, with a ramp rate of 0.04 °C per second and 25 acquisitions. In each run, a dilution series of 0 (unmethylated EpiTect Control DNA, Qiagen), 3.125, 6.25, 12.5, 25, 50 (fully methylated templates in fully unmethylated background), and 100 % (in vitro methylated DNA (IVD)) was included in order to test the sensitivity of the assay. Using the LightCycler 96 software, the melting curves were normalized relative to two normalization regions before and after the major fluorescence decrease. The methylation profile was evaluated for each sample by comparing fluorescence at the melting point against the value of fluorescence of the negative control (unmethylated DNA). Of the 241 samples analyzed, two samples (0.8 %) did not show a result.

### Data analysis

For direct-MSP and nested-MSP, we considered a sample as methylated if a clear band for the methylation-specific amplicon was visible by gel electrophoresis. We considered a weak methylation-specific amplicon as methylation positive only if the intensity of the amplicon was the same as for the amplicon generated with unmethylated primers. When in doubt, MSPs were repeated twice and the result of two out of three MSPs was considered final. Analyses of pyrosequencing results are more complex, as different thresholds for positivity can be defined. For each sample, we calculated the mean methylation percentage for six analyzed CpG dinucleotides. As we were interested in the effect of different thresholds for positivity, we analyzed our results with thresholds for positivity at 5, 10, 15, 20, and 25 %. MS-HRM has no single CpG dinucleotide resolution, and therefore, percentages of methylation cannot directly be converted from the melting curves. Therefore, within each assay run, we included a dilution series of controls with theoretically different methylation rates (unmethylated, 3.125, 6.25, 12.5, 25, and 50 % methylated and fully methylated DNA). Using the melting profile of the dilution series, we considered a sample as methylated if the melting curve deviated as the curve of the 3.125 % dilution or higher.

The *RET* promoter CpG island methylation frequency was presented for direct-MSP, nested-MSP, pyrosequencing, and MS-HRM as a percentage: the number of methylated samples divided by number of successfully amplified samples. In addition, for each assay, the overlap of methylation between techniques was determined. Therefore, we used the total number of methylated samples by direct-MSP, nested-MSP, pyrosequencing, and MS-HRM and compared them in a 2 × 2 table using SPSS.

To compare the clinical sensitivity and specificity of direct-MSP, nested-MSP, pyrosequencing, and MS-HRM, we computed clinical sensitivities and clinical specificities among deceased patients and subjects still alive, respectively, using overall mortality for the total follow-up period of up to 8 years as the standard. Clinical sensitivities and specificities were calculated for all assays as follows: we defined in the deceased patient group, the number of subjects (a) where the test was positive (true-positive) or (b) negative (false-negative) as well as the number of patients who are still alive but where the test was positive (c, false-positive) or negative (d, true-negative), using the formulas a/(a + b) and d/(c + d), respectively.

For survival analysis, overall survival (OS) was defined as time from cancer diagnosis until death of all causes. Three- and five-year survival analyses were performed for each methylation technique. Kaplan-Meier curves and log-rank tests were used to estimate the influence of methylation on overall survival. Hazard ratios (HR) and corresponding 95 % confidence intervals (CIs) were estimated using an unadjusted Cox proportional hazard model as previously described [27].

## Additional file

Additional file 1: Table S1. Primer sequences and conditions. Primer pairs were designed near the putative transcriptional start site. (DOCX 17 kb)

## References

[1] Imperiale TF, Ransohoff DF, Itzkowitz SH, Levin TR, Lavin P, Lidgard GP (2014). Multitarget stool DNA testing for colorectal-cancer screening. N Engl J Med.

[2] Ahlquist DA (2015). Multi-target stool DNA test: a new high bar for noninvasive screening. Dig Dis Sci.

[3] Church TR, Wandell M, Lofton-Day C, Mongin SJ, Burger M, Payne SR (2014). Prospective evaluation of methylated SEPT9 in plasma for detection of asymptomatic colorectal cancer. Gut.

[4] Stewart GD, Van Neste L, Delvenne P, Delree P, Delga A, McNeill SA (2013). Clinical utility of an epigenetic assay to detect occult prostate cancer in histopathologically negative biopsies: results of the MATLOC study. J Urol.

[5] Partin AW, Van Neste L, Klein EA, Marks LS, Gee JR, Troyer DA (2014). Clinical validation of an epigenetic assay to predict negative histopathological results in repeat prostate biopsies. J Urol.

[6] Renard I, Joniau S, van Cleynenbreugel B, Collette C, Naome C, Vlassenbroeck I (2010). Identification and validation of the methylated TWIST1 and NID2 genes through real-time methylation-specific polymerase chain reaction assays for the noninvasive detection of primary bladder cancer in urine samples. Eur Urol.

[7] McShane LM, Altman DG, Sauerbrei W, Taube SE, Gion M, Clark GM (2005). REporting recommendations for tumor MARKer prognostic studies (REMARK). Nat Clin Pract Urol.

[8] Draht MX, Riedl RR, Niessen H, Carvalho B, Meijer GA, Herman JG (2012). Promoter CpG island methylation markers in colorectal cancer: the road ahead. Epigenomics.

[9] Simon RM, Paik S, Hayes DF (2009). Use of archived specimens in evaluation of prognostic and predictive biomarkers. J Natl Cancer Inst.

[10] Begley CG, Ioannidis JP (2015). Reproducibility in science: improving the standard for basic and preclinical research. Circ Res.

[11] Ioannidis JP (2014). How to make more published research true. PLoS Med.

[12] Buffart TE, Overmeer RM, Steenbergen RD, Tijssen M, van Grieken NC, Snijders PJ (2008). MAL promoter hypermethylation as a novel prognostic marker in gastric cancer. Br J Cancer.

[13] Cleven AH, Derks S, Draht MX, Smits KM, Melotte V, Van Neste L (2014). CHFR promoter methylation indicates poor prognosis in stage II microsatellite stable colorectal cancer. Clin Cancer Res.

[14] Tanaka M, Chang P, Li Y, Li D, Overman M, Maru DM (2011). Association of CHFR promoter methylation with disease recurrence in locally advanced colon cancer. Clin Cancer Res.

[15] Hughes LA, Melotte V, de Schrijver J, de Maat M, Smit VT, Bovee JV (2013). The CpG island methylator phenotype: what’s in a name?. Cancer Res.

[16] van Vlodrop IJ, Niessen HE, Derks S, Baldewijns MM, van Criekinge W, Herman JG (2011). Analysis of promoter CpG island hypermethylation in cancer: location, location, location!. Clin Cancer Res.

[17] Quillien V, Lavenu A, Karayan-Tapon L, Carpentier C, Labussiere M, Lesimple T (2012). Comparative assessment of 5 methods (methylation-specific polymerase chain reaction, MethyLight, pyrosequencing, methylation-sensitive high-resolution melting, and immunohistochemistry) to analyze O6-methylguanine-DNA-methyltranferase in a series of 100 glioblastoma patients. Cancer.

[18] Herman JG, Graff JR, Myohanen S, Nelkin BD, Baylin SB (1996). Methylation-specific PCR: a novel PCR assay for methylation status of CpG islands. Proc Natl Acad Sci U S A.

[19] Kristensen LS, Mikeska T, Krypuy M, Dobrovic A (2008). Sensitive Melting Analysis after Real Time- Methylation Specific PCR (SMART-MSP): high-throughput and probe-free quantitative DNA methylation detection. Nucleic Acids Res.

[20] Shaw RJ, Akufo-Tetteh EK, Risk JM, Field JK, Liloglou T (2006). Methylation enrichment pyrosequencing: combining the specificity of MSP with validation by pyrosequencing. Nucleic Acids Res.

[21] Claus R, Wilop S, Hielscher T, Sonnet M, Dahl E, Galm O (2012). A systematic comparison of quantitative high-resolution DNA methylation analysis and methylation-specific PCR. Epigenetics.

[22] Uhlmann K, Brinckmann A, Toliat MR, Ritter H, Nurnberg P (2002). Evaluation of a potential epigenetic biomarker by quantitative methyl-single nucleotide polymorphism analysis. Electrophoresis.

[23] Colella S, Shen L, Baggerly KA, Issa JP, Krahe R (2003). Sensitive and quantitative universal pyrosequencing methylation analysis of CpG sites. Biotechniques.

[24] Tost J, Gut IG (2007). DNA methylation analysis by pyrosequencing. Nat Protoc.

[25] Wojdacz TK, Dobrovic A (2007). Methylation-sensitive high resolution melting (MS-HRM): a new approach for sensitive and high-throughput assessment of methylation. Nucleic Acids Res.

[26] Mikeska T, Candiloro IL, Dobrovic A (2010). The implications of heterogeneous DNA methylation for the accurate quantification of methylation. Epigenomics.

[27] Draht MX, Smits KM, Tournier B, Jooste V, Chapusot C, Carvalho B (2014). Promoter CpG island methylation of RET predicts poor prognosis in stage II colorectal cancer patients. Mol Oncol.

[28] Luo Y, Tsuchiya KD, Il Park D, Fausel R, Kanngurn S, Welcsh P (2013). RET is a potential tumor suppressor gene in colorectal cancer. Oncogene.

[29] van Roekel EH, Bours MJ, Breedveld-Peters JJ, Meijer K, Kant I, van den Brandt PA, et al. Light physical activity is associated with quality of life after colorectal cancer. Med Sci Sports Exerc. 2015. 10.1249/MSS.0000000000000698 25970666

[30] Barault L, Charon-Barra C, Jooste V, de la Vega MF, Martin L, Roignot P (2008). Hypermethylator phenotype in sporadic colon cancer: study on a population-based series of 582 cases. Cancer Res.

[31] Tournier B, Chapusot C, Courcet E, Martin L, Lepage C, Faivre J (2012). Why do results conflict regarding the prognostic value of the methylation status in colon cancers? The role of the preservation method. BMC Cancer.

[32] Derks S, Lentjes MH, Hellebrekers DM, de Bruine AP, Herman JG, van Engeland M (2004). Methylation-specific PCR unraveled. Cell Oncol.

