# Supplementary material for: Analysis of RET promoter CpG island methylation using methylation-specific PCR (MSP), pyrosequencing, and methylation-sensitive high-resolution melting (MS-HRM): impact on stage II colon cancer patient outcome
Source: Clin Epigenetics. 2016 Apr 26;8:44. doi: 10.1186/s13148-016-0211-8 (PMC4845472; doi:10.1186/s13148-016-0211-8)
Supplement: Additional file 1: Table S1. — Primer sequences and conditions. Primer pairs were designed near the putative transcriptional start site. (DOCX 17 kb) [file 13148_2016_211_MOESM1_ESM.docx]

Additional file 1: Table S1. Primer sequences and conditions.

| *Technique* | *Forward* | *Reverse* | *Tm (°C)* | *Cycles* |
| --- | --- | --- | --- | --- |
| Nested-MSP |  |  |  |  |
| Flank | GTTTTTTTTYGTATTTTATTYGTTTT | TTCCRAAACACTCAACRCTACTAC | 56 | 35 |
| U-specific | TTGGTTTTGTTTGGTTTATTTTTGGATTGTTTTTG | CTACACACCCTACTTCATCACAAAACTAAAACA | 66 | 30 |
| M-specific | GGTTTCGTTTGGTTTATTTTTGGATCGTTTTC | CTACTTCGATCGCGAAACTAAAACG | 66 | 30 |
| Direct-MSP |  |  |  |  |
| U-specific | TTGGTTTTGTTTGGTTTATTTTTGGATTGTTTTTG | CTACACACCCTACTTCATCACAAAACTAAAACA | 64 | 35 |
| M-specific | GGTTTCGTTTGGTTTATTTTTGGATCGTTTTC | CTACTTCGATCGCGAAACTAAAACG | 64 | 35 |
| Pyrosequencing |  |  |  |  |
| PCR | GGTTTAGTTTGGTTTATTTTTGGA | AATCTAACCCCCCCATCCCC* | 58 | 50 |
| Sequencing | GTTTAGTTTGGTTTATTTTTGGAT |  |  |  |
| MS-HRM | GTTTTTTTTYGTATTTTATTYGTTTT | TTCCRAAACACTCAACRCTACTAC | 56 | 70 |

*biotin labelled
